# Higher maternal thyroid resistance indices were associated with increased neonatal thyroid-stimulating hormone— analyses based on the Huizhou mother-infant cohort

**DOI:** 10.3389/fendo.2022.937430

**Published:** 2022-09-30

**Authors:** Shuyi Li, Yi Wu, Su-juan Zhang, Guoyi Li, Yu Tao Xiang, Wei-zhong Zhang, Wen-jing Pan, Wei-qing Chen, Yuan-tao Hao, Wen-hua Ling, Zhao-min Liu

**Affiliations:** ^1^ Guangdong Provincial Key Laboratory of Food, Nutrition and Health, School of Public Health, Sun Yat-sen University, Guangzhou, China; ^2^ School of Public Health, Sun Yat-sen University, Guangzhou, China; ^3^ Department of Clinical Nutrition, The First Huizhou Central Hospital, Huizhou, China; ^4^ Unit of Psychiatry, Department of Public Health and Medicinal Administration, University of Macao, Macao, Macau SAR, China; ^5^ Department of Pediatrics and Department of Health-care for Children Huizhou First Mother and Child Health-Care Hospital, Huizhou, China

**Keywords:** Maternal thyroid function, thyroid resistance indices, thyroid sensitivity, neonatal TSH, birth cohort

## Abstract

**Objectives:**

This study aimed to explore the relationship of maternal thyroid function and thyroid resistance parameters with neonatal thyroid-stimulating hormone (TSH).

**Methods:**

This work was a longitudinal study. Singleton pregnant women without a history of thyroid disorders were recruited in their first prenatal visit from October 2018 to June 2020. Maternal thyroid markers including TSH, free triiodothyronine (FT3), free thyroxine (FT4), and neonatal TSH were tested in the clinical laboratory of the hospital by electrochemiluminescence immunoassay. Thyroid resistance indices including Thyroid Feedback Quantile-based Index (TFQI), TSH index (TSHI), and thyrotroph T4 resistance index (TT4RI) were estimated in accordance with maternal FT4 and TSH levels. Multivariable linear and logistic regression was applied to explore the associations of maternal thyroid indices with infantile TSH level.

**Results:**

A total of 3,210 mothers and 2,991 newborns with valid TSH data were included for analysis. Multivariable linear regression indicated that maternal thyroid variables were significantly and positively associated with neonatal TSH levels with standardized coefficients of 0.085 for TSH, 0.102 for FT3, 0.100 for FT4, 0.076 for TSHI, 0.087 for TFQI, and 0.089 for TT4RI (all P < 0.001). Compared with the lowest quartile, the highest quartile of TSHI [odds ratio (OR) = 1.590, 95% CI: 0.928–2.724; P_trend_ = 0.025], TFQI (OR = 1.746, 95% CI: 1.005–3.034; P_trend_ = 0.016), and TT4RI (OR = 1.730, 95% CI: 1.021–2.934; P_trend_ = 0.030) were significantly associated with an increased risk of elevated neonatal TSH (>5 mIU/L) in a dose–response manner.

**Conclusion:**

The longitudinal data demonstrated that maternal thyroid resistance indices and thyroid hormones in the first half of gestation were positively associated with neonatal TSH levels. The findings offered an additionally practical recommendation to improve the current screening algorithms for congenital hypothyroidism.

## Introduction

Maternal thyroid function is critical for fetal and neonatal growth and neurodevelopment. Thyroid hormone (TH) deficiency or excess during embryonic development results in profound and permanent defects, and even transient hypothyroidism could cause adverse neurologic outcomes in a newborn (1). Congenital hypothyroidism (CH), a deficiency of TH at birth, is the most common and treatable cause of intellectual disability requiring immediate identification and treatment (1). The incidence of CH varied from 1:2,000 to 1:4,000 in different areas and ethnic groups (2, 3). A neonatal screening program has been implemented worldwide since the 1970s (3). However, less than one-third of the world’s birth population has been screened for CH, and approximately 29,840 CH cases per year do not benefit from early detection and treatment (2). The economic burden of disability owing to CH thus remains a remarkable public health challenge.

Studies have reported that maternal thyroid disorders during pregnancy were associated with multiple adverse pregnancy outcomes (4). However, limited studies explored the relationship of maternal THs with newborn thyroid function. Pregnancy causes increased thyroid gland vascularity and renal iodine clearance and elevated iodine needs from the fetus (5). Fluctuations in maternal thyroxine metabolism could impair the maternal-fetal transfer of thyroxine even in the euthyroid status (6). The reduced fetal thyroxine could cause a disruption to the development of the pituitary-thyroid axis of newborns (5, 6). Neonatal thyroid-stimulating hormone (TSH) has been considered as the most sensitive for detecting primary CH (7) and a predictive biomarker for iodine status in the population level (8). Elevated neonatal TSH suggests insufficient supply of TH to the developing fetal brain, and thus it is the only measure in predicting brain damage due to iodine deficiency (9).

Circulating THs are maintained within the normal range by a feedback mechanism operated by the hypothalamic-pituitary-thyroid (HPT) axis. At targeted tissues or organs, the adequate efficacy of THs depends on the capability and availability of TH transporters, deiodinases, and nuclear TH receptors (10). In humans, genetic defects in TH signaling could lead to various defects in TH membrane transport, metabolism, or action, even if they may not alter the TH profile (10). Resistance to TH (RTH) is a syndrome in which the responsiveness of end organs to TH is reduced (11). In 2009, Jostel et al. (12) first proposed the calculation of TSH index (TSHI) for estimating the sensitivity of the pituitary to THs. A reversible acquired RTH caused by endogenous and exogenous factors (i.e., comorbidities, malnutrition, or polypharmacy) was reported to be more common than the rare congenital RTH (13). In the general population, a modest acquired RTH has been suggested to be associated with an elevated risk of metabolic disorders and received increasing attention in recent years (14, 15). Owing to the inverse feedback loop, TSH and TH are physiologically and inversely correlated. However, the coexistence of high T4 and TSH levels was reported in patients of metabolic disorders suggesting a certain resistance to TH (11, 15). THs changed dramatically during gestation especially in the first half of pregnancy (16). However, the association of maternal thyroid sensitivity with neonatal TSH levels has not been investigated before. Verifying this association during gestation may have essential significance for predicting neonatal TSH and facilitating early screening of CH.

Although maternal thyroid disorder is generally considered a risk factor for CH (17), few studies have been conducted among euthyroid pregnant women. Most of the previous reports had small sample sizes, and neonatal TSH was often determined within 2 days after birth, a moment at which the association could be affected by a neonatal TSH surge (18). In addition, observational studies from iodine-sufficient regions were limited (9). Therefore, an ongoing longitudinal study of Huizhou mother-infant cohort was used to explore the associations of maternal thyroid function and thyroid resistance indices during early and mid-pregnancy with a subsequent risk of infantile hypothyroidism in an iodine-sufficient area of South China.

## Methods

### Study design and participant recruitment

This work was a longitudinal study conducted from October 2018 to June 2020 in Huizhou, a coastal city in Guangdong Province, South China. Singleton pregnant women aged 18–47 years were enrolled during their first antenatal visit. Women with known pre-pregnancy thyroid disorders or medications for thyroid treatment were excluded. Ethical approval was obtained from the Ethical Research Committee of Huizhou First Mother and Child Hospital. Written informed consents were signed by all participants before enrollment.

A total of 3,210 Chinese pregnant women and 2,991 paired newborns with valid TSH data were included for analysis. Personal information including sociodemographics, family and medical history, medication usages, dietary habits, and other lifestyle factors (i.e., smoking, alcohol drinking, and physical activity) was collected by the trained research staff *via* face-to-face interview during pre- and early pregnancy using a pretested questionnaire. Pre-pregnancy body mass index (BMI) was estimated on the basis of self-reported pre-pregnancy weight (kg) and measured height (m). Routine biochemical testing data including maternal thyroid markers in the first prenatal visit, plasma fasting and post-challenge glucose levels during mid-trimester, and neonatal TSH were all retrieved from the Hospital Information and Management System.

### Maternal and infantile thyroid testing

Maternal blood samples were collected after 8-12h of overnight fasting during the gestational weeks of 10–28. The specimens were centrifuged for 10 min at 3,000 r/min within 2 h after collection. Thyroid markers including TSH (mIU/L), free triiodothyronine (FT3, pmol/L), and free thyroxine (FT4, pmol/L) were routinely tested in the certified clinical laboratory of the hospital by electrochemiluminescence immunoassay (Roche Diagnostics, Indianapolis, IN, USA) on the Roche Cobas Elesys 602 Analyzer. Thyroid antibodies including antibodies of thyroperoxidase (TPOAb), TSH receptor (TRAb), and anti-thyroglobulin (TGAb) were further analyzed by immunoassay if the above TH levels were abnormal. Antibodies were regarded as positive when greater than 34 IU/L for TPOAb, 1.75 IU/L for TRAb, and 115 IU/L for TGA. All of the intra- and inter-assay coefficients of variation (CVs) for thyroid biomarkers were less than 10%.

FT3-to-FT4 ratio (FT3/FT4), a proxy of deiodinase activity, was calculated. Thyroid sensitivity was evaluated by thyrotroph T4 resistance index (TT4RI), TSHI, and Thyroid Feedback Quartile-based Index (TFQI) in accordance with the following formulas (19): TT4RI = FT4 (pmol/L) × TSH (mIU/L); TSHI = ln TSH (mIU/L) + 0.1345 × FT4 (pmol/L); TFQI = cdf FT4-(1-cdf TSH), where Cdf denoted cumulative distribution function (19, 20). The above thyroid resistance indices were calculated on the basis of maternal FT4 and TSH levels or their distributions, reflecting the sensitivity of the thyrotrophs to the feedback regulation by thyroid hormones. TSHI defines the maximum possible TSH response in the FT4-uninhibited state at a theoretical FT4 value of 0. TFQI was a new index of thyroid resistance that is based on the empirical joint distribution of FT4 and TSH, and it exhibited the difference between FT4 quantile and the reversed TSH quantile. The index focused on deviations from normality with the advantage of unsusceptible to extreme values even in cases of thyroid dysfunction.

Neonatal TSH levels, as a part of national screening project, were routinely tested by a chemiluminescence method using dried blood spot (DBS) by heel prick from newborns within 3–7 days after birth. The sensitivity of the TSH assay was 0.002 mIU/L. Neonates with TSH above 5 mU/L were considered at risk of thyroid dysfunction and suspicious for primary CH. They could be recalled for a second DBS collection and close follow-up.

### Establishment of reference intervals for maternal thyroid markers

In 2017, the American Thyroid Association recommended using trimester and assay-specific reference intervals in the local population for the diagnosis of thyroid disorders to ensure diagnostic accuracy and validity (21). Thus, 602 pregnant women who had been confirmed with negative TPOAb were enrolled in the present study to determine the reference intervals of thyroid markers. Women with normal TSH and FT4 (2.5th–97.5th percentile) were classified as euthyroid. The normal reference ranges in the first trimester were 0.01–3.76 mIU/L for TSH and 12.59–23.05 pmol/L for FT4. Overt or subclinical hypothyroidism was defined as TSH above 3.76 mIU/L with either normal or low FT4 (<12.59 pmol/L). Isolated hypothyroxinemia (IH) was defined as normal TSH in conjunction with a lower FT4.

### Statistical analysis

Statistical analyses were conducted using SPSS 21.0 software. Thyroid parameters including thyroid function markers (THs of FT3, FT4, and TSH) and thyroid resistance indices (TT4RI, TSHI, and TFQI) were treated as continuous and categorical variables (quartiles). Multivariable linear and logistic regression was adopted to explore the association of maternal thyroid function and thyroid resistance indices with neonatal TSH levels. A linear trend was tested by treating the ordinal value of each quartile as a continuous variable in the regression models. Potential confounders were selected on the basis of the results of univariate analyses, biological mechanisms or factors related with the outcomes, or the exposure of interest. Repeated analysis was conducted using Z-score-transformed thyroid markers as continuous variables to estimate the risk of elevated neonatal TSH level (>5 mIU/L) with 1 SD change of thyroid variables. A general linear model was additionally applied to compare the adjusted neonatal TSH levels among quartiles of thyroid parameters.

Sensitivity analyses were conducted to verify if the association remained similar 1) among women of euthyroid status (n = 2,916); 2) by exclusion of infants of low birth weight (n = 147) or preterm (n = 129), as they may have a delayed surge of TSH (22); and 3) by exclusion of women with any positive thyroid antibody (n = 18). Subgroup analyses were additionally explored by multivariable linear regression to testify the results’ consistency with stratification by maternal age (<40 vs. ≥40 years), BMI (<24.0 vs. ≥24 kg/m^2^), parity (null vs. ≥1), GDM status (yes vs. no), measuring time of THs (first vs. second trimester), maternal weight gain during gestation (<15 kg vs. ≥15 kg), infants’ gender (male vs. female), delivery modes (vaginal vs. C-section), and delivery time (<37 vs. ≥37 weeks).

## Results

**Figure 1:**
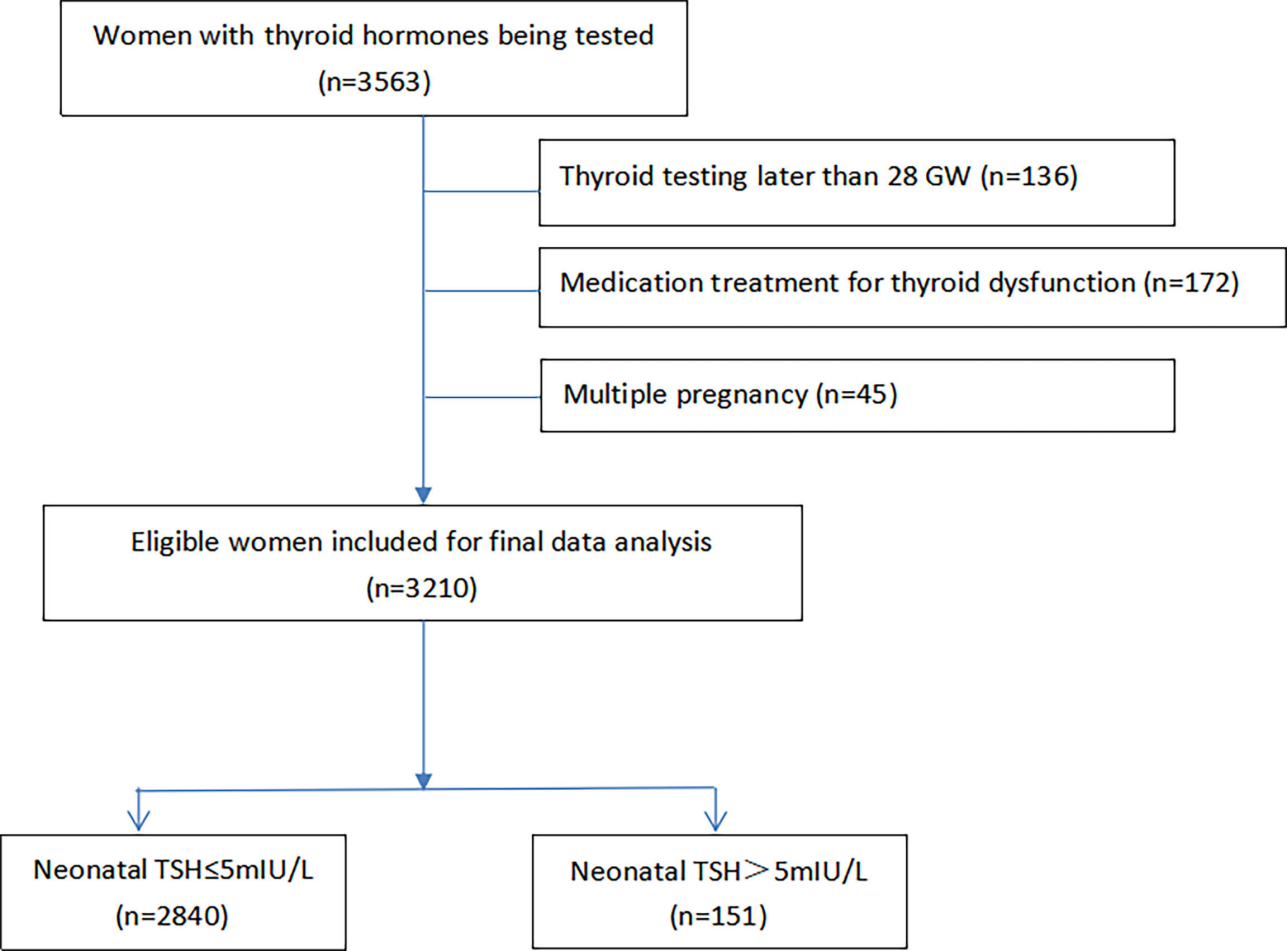
GW, gestational weeks; TSh, thyroid stimulating hormone. Among 3211 women of singleton pregnancy included for data analysis, 2991 neonatal TSH data were available from the Hospital information System.

After women who had multiple pregnancies, existing thyroid disorders, or with thyroid testing later than 28 gestation weeks were excluded, a total of 3,210 women were included for analysis (**Figure 1**). Among the 2,991 newborns with valid neonatal TSH levels, 151 (5.0%) had TSH levels above 5 mIU/L. The maternal basic characteristics and selected risk factors were indicated in **Table 1**. The average age of women was 28.4 ± 4.3 years with a pre-pregnancy BMI of 21.1 ± 3.4 kg/m^2^ and 45.6% multiparity. The multivariable linear regression results (**Table 2**) indicated that the maternal thyroid variables including THs and thyroid resistance indices were significantly and positively associated with neonatal TSH levels with standardized coefficients (β) of 0.085 for TSH, 0.102 for FT3, 0.100 for FT4, 0.076 for TSHI, 0.087 for TFQI, and 0.089 for TT4RI (all P < 0.001).

**Table 1:** Basic characteristics and birth outcomes of pregnant women, Huizhou mother-infant cohort 2018–2020 (n = 3,210).

Participants characteristics	n = 3,210
Maternal age (years)	28.4 ± 4.3
Education above high school, n (%)	1,829 (57.1)
Pre-pregnancy BMI (kg/m^2^)	21.1 ± 3.4
Weight gain during pregnancy (kg)	13. 7 ± 5.2
Gravity	2.1 ± 1.2
Parity ≥1, n (%)	1,344 (45.6)
Hormone usage, n (%)	723 (23.0)
Family history of first-degree relatives	
Thyroid disorders, n (%)	164 (5.1)
Type 2 diabetes, n (%)	267 (8.9)
Drinking, n (%)	124 (3.9)
Smoking, n (%)	30 (0.9)
Passive smoking, n (%)	1,797 (56.2)
Physical activity <30 min/day, n (%)	1,002 (35.0)
GDM during pregnancy, n (%)	567 (17.7)
Measuring time of thyroid hormones (gestational weeks)	13.0 ± 1.9
Delivery weeks (weeks)	39.2 ± 1.3
Delivery mode, n (%)	
Cesarean delivery or forceps	912 (28.4)
Vaginal delivery	2,299 (71.6)
Infant gender, n (%)	
Male	1,709 (53.2)
Female	1,501 (46.7)
Birth weight (kg)	3.16 ± 0.42
Birth length (cm)	49.9 ± 1.8
	n = 2,991
Neonatal TSH (mIU/L, median and IQR)	1.78 (1.08, 2.79)
Neonatal TSH ≥5 mIU/L	151 (5.0)

Data were expressed as mean ± standard deviation (SD) for continuous variables and n (%) for categorical variables.

TSH, thyroid-stimulating hormone (mIU/L); BMI, body mass index; GDM, gestational diabetes mellitus; IQR, interquartile range.

Smoking or drinking was defined as any smoking or alcohol drinking at least once during pre- or early pregnancy. Hormone usage was defined as usage of any hormonal medications including steroid or sex hormones during pre- or early pregnancy. GDM diagnosis was conducted during 24–28 gestational weeks based on the recommendation of the International Association of Diabetes and Pregnancy Study Groups (IADPSG) with 0-, 1-, and 2-h glucose levels above respective cutoffs: 0 h, ≥5.1 mmol/L; 1 h, ≥10.0 mmol/L; or 2 h, ≥8.5 mmol/L.

**Table 2:** Multivariable linear regression on the associations of maternal thyroid function and thyroid resistance indices with infantile TSH level, Huizhou mother-infant cohort (n = 3,210).

Independent variables	Dependent variable: neonatal TSH (mIU/L)			P value
	Unstandardized Coefficients β (95% CI)	Standardized Coefficients β	Adjusted R^2^	
**Thyroid function markers**				
TSH (mIU/L)				
Model 1 (Crude)	0.051 (0.019, 0.082)	0.057	0.007	0.002
Model 2 (Adjusted)	0.121 (0.067, 0.176)	0.085	0.022	<0.001
FT3 (pmol/L)				
Model 1 (Crude)	0.159 (0.115, 0.203)	0.129	0.016	<0.001
Model 2 (Adjusted)	0.175 (0.109, 0.241)	0.102	0.032	<0.001
FT4 (pmol/L)				
Model 1 (Crude)	0.051 (0.036, 0.066)	0.120	0.014	<0.001
Model 2 (Adjusted)	0.049 (0.029, 0.068)	0.100	0.030	<0.001
FT3/FT4				
Model 1 (Crude)	-0.296 (-1.670, 1.079)	-0.008	0	0.673
Model 2 (Adjusted)	-0.756 (-2.348, 0.836)	-0.020	0.016	0.352
**Thyroid resistance indices**				
TSH Index (TSHI)				
Model 1 (Crude)	0.129 (0.083, 0.175)	0.100	0.010	<0.001
Model 2 (Adjusted)	0.099 (0.049, 0.149)	0.076	0.024	<0.001
TFQI				
Model 1 (Crude)	0.485 (0.298, 0.671)	0.093	0.008	<0.001
Model 2 (Adjusted)	0.448 (0.243, 0.654)	0.087	0.023	<0.001
TT4RI				
Model 1 (Crude)	0.005 (0.002, 0.007)	0.070	0.008	<0.001
Model 2 (Adjusted)	0.008 (0.005, 0.012)	0.089	0.023	<0.001

Data were analyzed by multivariable linear regression by enter method with the adjusted covariates for model 2 including maternal age at pregnancy (years), parity, first-degree relatives with thyroid diseases (yes or no), education (less or equivalent to primary school, middle school, above high school), pre-pregnancy body mass index (kg/m^2^), body weight gain during pregnancy (kg), measuring time of thyroid hormones (gestational weeks), gestational diabetes mellitus (yes or no),delivery mode (Cesarean delivery/forceps, vaginal delivery), gestational weeks of delivery, gender of infant (male or female), and birth weight (kg).

TSH, thyroid-stimulating hormone; CI, confidence interval; FT3, free triiodothyronine; FT4, free thyroxine; TFQI, Thyroid Feedback Quantile-based Index; TT4RI, thyrotrophic T4 resistance index.

TFQI = cdfFT4- (1-cdfTSH); TSH index = lnTSH (mIU/L) + 0.1345 × FT4 (pmol/L); TT4RI = FT4 (pmol/L) × TSH (mIU/L).

When maternal and infantile thyroid variables were categorized, the results of multivariable logistic regression (**Table 3**) indicated that compared with the lowest quartile (reference), the women in the highest quartile of thyroid parameters had marginally or significantly increased risk of elevated neonatal TSH (>5 mIU/L) by 59.0% [odds ratio (OR) = 1.590, 95% CI: 0.928–2.724; P_trend_ = 0.025] for TSHI, 74.6% (OR = 1.746, 95% CI: 1.005–3.034; P_trend_ = 0.016) for TFQI, and 73.0% (OR = 1.730, 95% CI: 1.021–2.934; P_trend_ = 0.030) for TT4RI. With each 1 SD increase in maternal thyroid parameters, the risk of elevated neonatal TSH (>5 mIU/L) increased by 43.9% for maternal TSH (P = 0.009), 25.6% for maternal FT3 (P = 0.004), 21.9% for FT4 (P = 0.008), 25.2% for TSHI (P = 0.040), 24.9% for TFQI (P = 0.018), 28.2% for TT4RI (P = 0.009). Meanwhile, a lack of significance for FT3-to-FT4 ratio was found. The general linear model with the neonatal TSH level as the dependent variable also indicated (**Supplementary Table S3**) that thyroid variables in the highest quartile group exhibited significantly higher neonatal TSH levels than those in the lowest quartile, with the mean differences between the extreme quartiles (Q4–Q1) of 0.317 (95% CI: 0.138–0.497) mIU/L for TSH, 0.296 (95% CI: 0.118–0.473) for TSHI, 0.306 (95% CI: 0.126–0.487) for TFQI, and 0.326 (95% CI: 0.148–0.504) for TT4RI (all P ≤ 0.001).

**Table 3:** Odds ratios (ORs) and 95% confidence intervals (CIs) of neonatal TSH levels >5 mIU/L by quartiles of maternal thyroid function and thyroid resistance indices, Huizhou mother-infant cohort.

Maternal thyroid markers	Odds ratios and 95% confidence intervals of neonatal TSH >5 mIU/L						
	Q1	Q2	Q3	Q4	P_trend_	Adjusted OR (95% CI) for 1SD Increase	P value
**Thyroid function**							
**TSH (mIU/L), n**	792	814	810	794			
Median (min~)	0.189 (0.000~)	0.867 (0.560~)	1.470 (1.140~)	2.558 (1.910~79.810)			
Crude OR (95% CI)	1 [Reference]	1.291 (0.776, 2.149)	1.416 (0.858, 2.339)	1.860 (1.149, 3.009)	0.010		
Adjusted OR (95% CI)	1 [Reference]	1.223 (0.710, 2.107)	1.259 (0.729, 2.172)	1.671 (0.993, 2.814)	0.055	1.439 (1.095, 1.890)	0.009
**FT3 (pmol/L), n**	809	805	809	787			
Median (min~max)	4.160 (2.840~)	4.600 (4.420~)	5.010 (4.800~)	5.570 (5.230, 43.670)			
Crude OR (95% CI)	1 [Reference]	1.096 (0.704, 1.707)	0.673 (0.409, 1.109)	1.066 (0.687, 1.669)	0.739		
Adjusted OR (95% CI)	1 [Reference]	1.062 (0.652, 1.729)	0.695 (0.407, 1.188)	1.048 (0.632, 1.738)	0.747	1.256 (1.075, 1.469)	0.004
**FT4 (pmol/L), n**	801	806	805	798			
Median (min~max)	14.320 (8.330~)	15.980 (15.250~)	17.410 (16.690~)	19.740 (18.300, 100.000)			
Crude OR (95% CI)	1 [Reference]	1.048 (0.658, 1.667)	1.053 (0.663, 1.670)	1.010 (0.633, 1.612)	0.963		
Adjusted OR (95% CI)	1 [Reference]	1.063 (0.631, 1.793)	1.051 (0.617, 1.791)	1.210 (0.711, 2.060)	0.509	1.219 (1.052, 1.413)	0.008
**FT3/FT4, n**	797	811	807	795			
Median (min~max)	0.244 (0.099~)	0.274 (0.260~)	0.299 (0.286~)	0.339 (0.315, 0.572)			
Crude OR (95% CI)	1 [Reference]	0.934 (0.595, 1.466)	0.664 (0.406, 1.086)	1.075 (0.692, 1.669)	0.924		
Adjusted OR (95% CI)	1 [Reference]	0.912 (0.559, 1.489)	0.624 (0.362, 1.074)	0.942 (0.559, 1.588)	0.533	1.004 (0.827, 1.217)	0.971
**Thyroid resistance indices**							
**TSHI, n**	795	802	818	795			
Median (min~max)	0.757 (-5.910~)	2.066 (1.690~)	2.593 (2.340~)	3.155 (2.840, 8.150)			
Crude OR (95% CI)	1 [Reference]	1.224 (0.709, 2.115)	2.227 (1.360, 3.648)	1.867 (1.123, 3.104)	0.002		
Adjusted OR (95% CI)	1 [Reference]	1.088 (0.612, 1.934)	1.858 (1.104, 3.125)	1.590 (0.928, 2.724)	0.025	1.252 (1.010, 1.553)	0.040
**TFQI, n**	798	803	802	807			
Median (min~max)	-0.373 (-0.874~)	-0.081 (-0.198~)	0.073 (0.002~)	0.370 (0.191, 0.931)			
Crude OR (95% CI)	1 [Reference]	1.436 (0.850, 2.424)	1.994 (1.216, 3.270)	1.747 (1.055, 2.890)	0.015		
Adjusted OR (95% CI)	1 [Reference]	1.239 (0.702, 2.186)	2.032 (1.191, 3.467)	1.746 (1.005, 3.034)	0.016	1.249 (1.038, 1.503)	0.018
**TT4RI, n**	795	803	814	798			
Median (min~max)	3.377 (0.003~)	14.054 (9.551~)	23.873 (18.734~)	40.989 (30.415, 951.335)			
Crude OR (95% CI)	1 [Reference]	1.377 (0.816, 2.324)	1.753 (1.061, 2.895)	1.921 (1.169, 3.155)	0.006		
Adjusted OR (95% CI)	1 [Reference]	1.234 (0.707, 2.164)	1.478 (0.862, 2.533)	1.730 (1.021, 2.934)	0.030	1.282 (1.065, 1.542)	0.009

Data were analyzed by both univariate and multivariable logistic regression models. For multivariable regression models, the adjusted covariates were included by enter method including maternal age of pregnancy (years), education (less or equivalent to primary school, middle school, above high school), parity, first-degree relatives with thyroid disorders (yes or no), maternal pre-pregnancy body mass index (kg/m2), body weight gain during pregnancy (kg), measuring time of thyroid hormones (gestational weeks), gestational diabetes mellitus (yes or no), delivery modes (Cesarean delivery or forceps, vaginal delivery), gestational weeks of delivery, gender of infant (male or female), and birth weight (kg).

Thyroid parameters were additionally treated as continuous variables and transformed as Z-scores in logistic regression models for estimation of the risk of elevated neonatal TSH (>5 mIU/L) by 1 SD change of thyroid markers. One extreme value in Q4 of TSH and TT4RI was replaced by the median of Q4.

OR, odds ratio; CI, confidence interval; TSH, thyroid-stimulating hormone; FT3, free triiodothyronine; FT4, free thyroxine; TFQI, Thyroid Feedback Quantile-based Index; TT4RI, thyrotrophic T4 resistance index.

TFQI = cdfFT4- (1-cdfTSH); TSH index = lnTSH (mIU/L) + 0.1345 × FT4 (pmol/L); TT4RI = FT4 (pmol/L) × TSH (mIU/L).

Sensitivity analyses among euthyroid women (n = 2,916) or by exclusion of infants of preterm (<37 gestational weeks, n = 129) and low birth weight (birth weight <2.5 kg, n = 147) indicated similar findings (data not shown). Pregnant women with thyroid dysfunction including clinical and subclinical hypothyroidism and overt hyperthyroidism had significantly increased neonatal TSH levels (**Supplementary Table S1**). The neonatal TSH levels were significantly higher in male newborns or infants of vaginal delivery (**Supplementary Table S2** **).** Subgroup analyses by multivariable linear regression suggested more evident associations between maternal thyroid parameters and neonatal TSH levels, being observed among women aged less than 40 years or with lower BMI or weight gain, non-GDM status, TH testing at first trimester, and vaginal delivery and giving birth to male or full-term babies (**Supplementary Table S4**).

## Discussion

The analysis indicated that higher maternal THs and thyroid resistance indices were significantly and positively associated with increased infantile TSH at birth. To our knowledge, this study was the first to explore the relationship of maternal thyroid sensitivity with neonatal thyroid function. The findings implied an additionally practical recommendation to improve the current screening algorithms for CH. Mothers with reduced thyroid sensitivity during pregnancy showed an increased risk of neonatal TSH elevation. Additional evaluation of thyroid sensitivity based on routine thyroid assays of TSH and FT4 during early gestation could facilitate early CH screening and protect newborns from late diagnosis and management of CH.

Abnormal neonatal thyroid function after screening is required for close follow-up before initiation of treatment. Delayed diagnosis and treatment of CH could lead to the most severe outcome of intellectual disability. Neonatal TSH screening has been shown to be more specific, sensitive, and cost-effective in the diagnosis of CH than T4 screening due to the high false-positive rate of T4 in low-birth weight and premature infants (23). Neonatal TSH levels could be affected by a range of stress-related maternal and neonatal factors including age at pregnancy, maternal thyroid function and antibodies, delivery time and mode, infant gender, birth weight and height, and even the time of DBS collection after delivery (23). The results of the present study were in line with those of a previous report (22) that showed that neonatal TSH was significantly lower in female, preterm, and low-birth weight infants. Premature newborns exhibited delayed elevation in TSH possibly due to an immaturity of the HPT axis and/or the low storage of iodine in these infants (22).

Pregnancy has a significant effect on HPT physiology due to increased plasma volume and human chorionic gonadotropin (HCG) level and enhanced deiodinase activity and urinary iodine clearance (24). The pattern of thyroid markers in pregnancy suggests physiologic adaptations to optimize maternal thyroid status for fetal development (24). The findings were consistent with those of previous reports, which revealed that maternal FT3, FT4, and TSH were positively associated with offspring blood or cord TSH level after delivery (4, 25–27) (28) but not all (29). Several shared factors between mother and infant, such as genetics and nutrition (e.g., iodine status), are known to influence thyroid parameters (30). This influence could be due to that the high estrogen and HCG levels during pregnancy could modify TSH release directly at the pituitary level (31). A significant association was not observed between maternal FT3/FT4 and neonatal TSH. The changes in FT3 and FT4 levels follow a parallel pattern, which may be related to the ratio of FT3 to FT4 remaining essentially unaltered (16) and exhibiting a small range between Q1 and Q4 in the data of the present study.

Although THs play essential roles in energy balance and weight control, the data suggested the positive associations of RTH indices with neonatal TSH levels. The associations were independent of pre-pregnant BMI, maternal GDM status, and weight gain during gestation. Homeostasis could be achieved in the thyroid system through the coordination of the negative feedback regulation by the HPT axis and a set of iodothyronine deiodinase and TH receptor in peripheral tissues (32). Reduced sensitivity to TH in peripheral tissues is often termed acquired resistance to TH (13). A range of physiological or pathological conditions such as aging (33), pregnancy (34), drugs or toxins (30), and intercurrent non-thyroidal illness (15) could lead to reduced sensitivity to TH *via* changing hormonal and autoimmune systems (29). Such impairment may not be picked up by assays of TH and TSH (33). The euthyroid status of women in pregnancy could be an adaptive response attempting to conserve energy and shut down unessential processes to ensure fetal growth and development (33). This adaptive change may be a beneficial response to reduce the metabolic rate (35). The subgroup analysis in the present study also suggested that the positive associations of thyroid resistance with neonatal TSH levels were more evident among women of less gestational weight gain or lower BMI, a hypometabolism status. The hypothesis on the adaptive response of neonatal TSH to a certain extent was supported by a report in congenital RTH mothers. The study (36) in RTH-β-affected mothers (mutations in the THR β gene, with high TH levels) reported significantly lower birth weight and suppressed neonatal TSH, suggesting that a notable excess of maternal TH could impair the embryogenesis of growing fetuses (the uncompensated status). However, another case study (36) reported that neonates born to asymptomatic RTH mothers were healthy, with normal growth scans and normal thyroid function tests at 1 week (37). Whether or not high levels of maternal TH could exert a toxic effect on fetal TSH or development remains controversial. The findings implicated the importance of a universal thyroid screening strategy during gestation, as the acquired RTHs in TH responsiveness are usually under normal ranges of plasma levels of TH and TSH.

Several mechanisms may explain the observed positive associations of maternal thyroid resistance indices with neonatal TSH levels. First, gestation implies an increased burden on the thyroid gland that could stimulate a higher production of THs, predisposing to thyroid resistance. Mothers with decreased TH sensitivity tended to have increased FT4 or TSH levels, which have a direct effect on fetus TSH level by placental transfer. The increased neonatal TSH may be a kind of neonatal resistance to TH, as described in wild-type children born from RTH mothers (38). Second, the decreased thyroid sensitivity could be due to the reduced TH receptor in target organs (39) that may induce a decrease in hormone action, thereby increasing plasma TSH and causing a condition of peripheral RTH (39). The reversible acquired RTH status may stimulate the homeostatic compensation mechanism, which is an adaptation process (13, 29). A slightly increased TSH may protect the fetus from the toxic effect of maternal TH excess.

The study has several strengths. First, it was the first study exploring the associations of maternal thyroid resistance indices with infantile thyroid function. Second, this population-based prospective study had a relatively large sample size and tested a number of biomedical markers during the women’s first antenatal visit. In addition, the neonatal TSH level was determined within 3–7 days after birth, thus minimizing the false-positive high TSH values due to the neonatal TSH surge (7). Moreover, women with prior thyroid disorders before pregnancy were excluded to avoid potential biases. Third, all of the biochemical measurements were conducted in the same clinical laboratory under strict quality control to ensure the results’ validity. Fourth, the trimester- and population-specific reference intervals for THs were established, allowing for accurate classification of thyroid disorders. Finally, for statistical analysis, maternal and infantile thyroid markers were treated as continuous and categorical variables to strengthen the validity of the findings and elaborate different implications from clinical and public health perspectives. A number of sensitivity and subgroup analyses were additionally performed to confirm the consistency of the findings under various subpopulations or scenarios.

Several limitations in this study should be acknowledged. First, thyroid antibodies were only tested among pregnant women with an abnormal thyroid function. Thus, the presence of thyroid autoimmunity may not be entirely excluded, although sensitivity analyses specifically among euthyroid women or by exclusion of women with positive thyroid antibodies (n = 18) showed similar findings with the original ones. Current studies regarding maternal thyroid autoimmunity with birth outcomes and neonatal thyroid function yielded mixed findings (40, 41), and the routine screening for thyroid autoimmunity among pregnant women needs more evidence for recommendation (41). Second, maternal iodine status during early gestation was not evaluated. The data indicated that 5.0% of neonates had TSH levels above 5 mIU/L, suggesting insufficient iodine in participants compared with the WHO criterion of ≤3% (42). Although living in a coastal city in South China with ample iodine-rich food supply, a considerable portion of pregnant women is still at risk of iodine deficiency due to increased demands from fetus, hormonal changes during gestation, and insufficient dietary intake due to morning sickness. In addition, the use of neonatal TSH for monitoring iodine status as a sensitive and reliable quantitative tool remains doubtful (43). Adopting a lower cut point for neonatal TSH may increase the number of false positives although improving the sensitivity (44). Third, neonatal T4 level was not tested because screening by testing both T4 and TSH is not cost-effective. The primary TSH test strategy has been adopted by most programs around the world due to the lower recall rate than that of primary T4 or combined T4 and TSH strategy (45). Fourth, the current data were from one hospital only. The findings may not be generalizable to other ethnicities or regional populations. Finally, as in other cohort analyses, potential residual confounding may not be entirely excluded.

## Conclusions

This study indicated that maternal THs and resistance indices in the first half of gestation were positively associated with neonatal TSH levels. The findings revealed the benefit of early CH screening *via* assessment of maternal thyroid sensitivity. Future research is needed to investigate whether the association could mediate adverse child neural development.

## Data availability statement

The raw data supporting the conclusions of this article will be made available by the authors, without undue reservation.

## Ethics statement

The studies involving human participants were reviewed and approved by ethics committee of Huizhou first maternal and Child Health Hospital. The patients/participants provided their written informed consent to participate in this study.

## Author contributions

Z-mL and SL conceptualized the topic, made results explanation and drafted the manuscript; SL and GL conducted data collection, data polishing and analysis. W-jP coordinated the study investigation and approved data utility; other co-authors critically reviewed and commented on the manuscript. All authors contributed to the article and approved the submitted version.

## Funding

The research was funded by the “Hundred Talents Program” of Sun Yat-sen University (Grant NO: 51000-18841203) and the National Natural Science Foundation of China (NSFC, No.82073533).

## Acknowledgments

We are grateful to research members for their efforts in field investigation and data collection. We greatly appreciate the doctors and nurses in the Huizhou First Mother and Child Hospital for their keen assistance in facilitating participants’ recruitment and investigation and data coordination. We acknowledge the Department of Clinical Laboratory and Department of Hospital Information Management of Huizhou Hospital for providing great support in analysing bio-specimens and data utility. We acknowledge the research support from Guangdong Provincial Key Laboratory of Food, Nutrition and Health (W-hL), and Shengzhen Sanming Project (Y-tH and W-qC).

## Conflict of interest

The authors declare that the research was conducted in the absence of any commercial or financial relationships that could be construed as a potential conflict of interest.

## Publisher’s note

All claims expressed in this article are solely those of the authors and do not necessarily represent those of their affiliated organizations, or those of the publisher, the editors and the reviewers. Any product that may be evaluated in this article, or claim that may be made by its manufacturer, is not guaranteed or endorsed by the publisher.
