# Supplementary material for: Higher maternal thyroid resistance indices were associated with increased neonatal thyroid-stimulating hormone— analyses based on the Huizhou mother-infant cohort
Source: Front Endocrinol (Lausanne). 2022 Sep 30;13:937430. doi: 10.3389/fendo.2022.937430 (PMC9561092; doi:10.3389/fendo.2022.937430)
Supplement: Supplementary file 1 [file Table_1.docx]

**Supplemental Table 1 Neonatal TSH levels by different maternal thyroid conditions (n=2991).**

|  | **Neonatal TSH levels (mIU/L)** | **Neonatal TSH > 5 mIU/L ，n(%)** |
| --- | --- | --- |
| **Euthyroid women (n=2721)** | 2.158±1.534 | 132 (4.8) |
| **Subclinical hypothyroidism (n=102)** | 2.766±2.784 | 11 (10.8) |
| **Overt hypothyroidism (n=6)** | 3.082±2.244 | 1 (12.5) |
| **Subclinical hyperthyroidism (n=24)** | 1.902±0.848 | 0 (0) |
| **Overt hyperthyroidism (n=47)** | 2.793±3.537 | 5 (10.6) |
| **Isolated hypothyroxinaemia (n=46)** | 1.821±1.408 | 2 (4.2) |
| **Undefined group (n=45)** | 1.806±0.987 | 0 (0) |
| **Total (n=2991)** | 2.178±1.636 | 151 (5.0) |

Among the 3210 eligible women (singleton pregnancy, thyroid testing before 28 gestational weeks and no medication treatment during pre-or early pregnancy) included for data analysis, 2991 had neonatal TSH data. The current diagnosis of thyroid dysfunction was based on the newly established cutoffs of thyroid hormones. Data were expressed as means±standard deviation for continuous variables and n (%) for categorical variables. Abbreviations: TSH, thyroid stimulating hormone.

**Supplemental Table 2 Neonatal TSH levels by different levels of maternal age,BMI, purity, maternal GDM status, neonatal gender, measuring time of thyroid testing, delivery mode and delivery week （n=3210）**

| **Subgroup variables** | **n** | **Neonatal TSH level (mIU/L)** | **P value** |
| --- | --- | --- | --- |
| **Maternal age (years)** |  |  | 0.822 |
| ＜40 | 3183 | 2.179±1.638 |  |
| ≥40 | 27 | 2.116±1.448 |  |
| **Maternal body weight gain(kg)** |  |  | 0.875 |
| ＜15 | 1779 | 2.163±1.604 |  |
| ≥15 | 1034 | 2.226±1.678 |  |
| **Maternal Pre-pregnancy BMI (kg/m^2^)** |  |  | 0.556 |
| ＜24 | 2654 | 2.190±1.634 |  |
| ≥24 | 554 | 2.112±1.633 |  |
| **Parity** |  |  | 0.100 |
| 0 | 1603 | 2.221±1.732 |  |
| ≥1 | 1344 | 2.136±1.534 |  |
| **Maternal GDM during pregnancy** |  |  | 0.662 |
| Yes | 573 | 2.189±1.647 |  |
| No | 2634 | 2.134±1.590 |  |
| **Measuring time of thyroid hormones** |  |  | 0.571 |
| 1^st^ trimester | 2013 | 2.165±1.626 |  |
| 2^nd^ trimester | 1197 | 2.200±1.653 |  |
| **Neonatal gender** |  |  | 0.001 |
| Male | 1709 | 2.306±1.758 |  |
| Female | 1500 | 2.034±1.474 |  |
| **Delivery modes** |  |  | 0.032 |
| Vaginal | 2299 | 2.276±1.653 |  |
| Cesarean or triceps | 911 | 1.938±1.567 |  |
| **Delivery week（gestational weeks）** |  |  | 0.109 |
| ＜37 | 129 | 1.838±1.423 |  |
| ≥37 | 3082 | 2.192±1.643 |  |

Women of singleton pregnancy，without medication treatment for thyroid disorders during pre-pregnancy and thyroid testing before 28 gestational weeks were included for analysis (n=3211). Data were expressed as means±standard deviation and the comparisons were made by student T-test.

**Supplemental Table 3. Comparison of neonatal TSH levels by quartiles of maternal thyroid markers using general linear model, Huizhou Birth cohort (n=3210)**

| Maternal thyroid markers | Dependent variable: Neonatal TSH (mIU/L) | | | | | | |
| --- | --- | --- | --- | --- | --- | --- | --- |
|  | Adjusted mean±standard error (SE) by quartiles of maternal thyroid markers | | | |  | Mean difference(95% CI) | |
|  | Q1 | Q2 | Q3 | Q4 | P for trend | Q4-Q1 | P value |
| Thyroid function markers |  |  |  |  |  |  |  |
| TSH (mIU/L) | 2.036±0.064 | 2.161±0.062 | 2.188±0.063 | 2.360±0.064 | 0.006 | 0.317（0.138，0.497） | 0.001 |
| FT3 (pmol/L) | 2.212±0.064 | 2.212±0.064 | 2.146±0.063 | 2.176±0.064 | 0.874 | -0.028（-0.210，0.154） | 0.763 |
| FT4 (pmol/L) | 2.143±0.064 | 2.194±0.064 | 2.192±0.063 | 2.217±0.064 | 0.760 | 0.101（-0.087，0.288） | 0.292 |
| FT3/FT4 | 2.231±0.066 | 2.225±0.063 | 2.140±0.063 | 2.149±0.066 | 0.598 | -0.093（-0.283，0.098） | 0.342 |
| Thyroid resistance indices |  |  |  |  |  |  |  |
| TSH index (TSHI) | 2.022±0.063 | 2.090±0.063 | 2.310±0.063 | 2.322±0.064 | 0.001 | 0.296（0.118，0.473） | 0.001 |
| TFQI | 2.010±0.064 | 2.077±0.063 | 2.355±0.064 | 2.303±0.064 | <0.001 | 0.306（0.126，0.487） | 0.001 |
| TT4RI | 2.036±0.064 | 2.107±0.063 | 2.234±0.063 | 2.368±0.064 | 0.002 | 0.326（0.148，0.504） | <0.001 |

Pregnant women of singleton, without medication treatment for thyroid disorders during pre-pregnancy and available thyroid testing before 28 gestational weeks were included for analysis (n=3211). Data were analyzed by general linear model and expressed as estimated marginal means and standard error for each quartile with covariates being adjusted including maternal age of pregnancy(y), education(less or equivalent to primary school, middle school, above high school), parity, 1st degree relatives of thyroid disorders(yes or no), maternal pre-pregnancy body mass index(kg/m2), body weight gain during pregnancy(kg),measuring time of thyroid hormones (gestational weeks),gestational diabetes mellitus(yes or no),delivery mode(cesarean delivery or forceps, vaginal delivery), gestational weeks of delivery, gender of infant(male or female), birth weight(kg). Pairwise comparisons were made by LSD method and reported mean difference and 95% CI between extreme quartiles (Q4-Q1).

Abbreviations: TSH, thyroid stimulating hormone (mIU/L); SE, standard error; CI, confidence interval; Q,quartile;FT3, free triiodothyronine (pmol/L); FT4,free thyroxine (pmol/L); TFQI, thyroid feedback quantile-based index; TT4RI,thyrotrophic T4 Resistance Index. TFQI=cdfFT4-(1-cdfTSH);TSH index=lnTSH (mIU/L) + 0.1345×FT4(pmol/L);TT4RI=FT4 (pmol/L)×TSH(mIU/L)

**Supplemental Table 4 Subgroup analyses by maternal age ,BMI, parity, maternal GDM), measuring trimester of thyroid hormones, neonatal gender, delivery mode and delivery week on the associations of maternal thyroid parameters and neonatal TSH levels by multivariable linear regression models (n=3210)**

|  |  | **FT3** |  | **FT4** |  | **TSH** |  | **TT4RI** |  | **TSHI** |  | **TFQI** |  |
| --- | --- | --- | --- | --- | --- | --- | --- | --- | --- | --- | --- | --- | --- |
|  | n | β | P | β | P | β | P | β | P | β | P | β | P |
| **Maternal age (y)** |  |  |  |  |  |  |  |  |  |  |  |  |  |
| ＜40 | 3184 | 0.145 | <0.001 | 0.135 | <0.001 | 0.052 | 0.006 | 0.064 | 0.001 | 0.093 | <0.001 | 0.091 | <0.001 |
| ≥40 | 27 | -0.239 | 0.197 | -0.159 | 0.474 | 0.137 | 0.477 | 0.127 | 0.523 | 0.281 | 0.130 | 0.045 | 0.824 |
| **Maternal BW gain(kg)** |  |  |  |  |  |  |  |  |  |  |  |  |  |
| ＜15 | 1779 | 0.186 | <0.001 | 0.168 | <0.001 | 0.040 | 0.117 | 0.052 | 0.040 | 0.084 | 0.001 | 0.100 | <0.001 |
| ≥15 | 1034 | -0.048 | 0.158 | -0.020 | 0.571 | 0.112 | 0.001 | 0.110 | 0.001 | 0.070 | 0.036 | 0.064 | 0.063 |
| **Maternal BMI (kg/m^2^)** |  |  |  |  |  |  |  |  |  |  |  |  |  |
| ＜24 | 2617 | 0.093 | <0.001 | 0.081 | <0.001 | 0.059 | 0.005 | 0.073 | <0.001 | 0.098 | <0.001 | 0.096 | <0.001 |
| ≥24 | 554 | 0.405 | <0.001 | 0.365 | <0.001 | -0.009 | 0.839 | -0.014 | 0.759 | 0.059 | 0.200 | 0.050 | 0.282 |
| **Parity** |  |  |  |  |  |  |  |  |  |  |  |  |  |
| 0 | 1603 | 0.158 | <0.001 | 0.134 | <0.001 | 0.038 | 0.199 | 0.050 | 0.090 | 0.091 | 0.002 | 0.090 | 0.003 |
| ≥1 | 1608 | 0.100 | 0.001 | 0.100 | 0.002 | 0.111 | <0.001 | 0.109 | <0.001 | 0.062 | 0.044 | 0.082 | 0.009 |
| **Maternal GDM** |  |  |  |  |  |  |  |  |  |  |  |  |  |
| No | 2635 | 0.157 | <0.001 | 0.150 | <0.001 | 0.052 | 0.013 | 0.066 | 0.002 | 0.106 | <0.001 | 0.097 | <0.001 |
| Yes | 573 | 0.038 | 0.410 | 0.027 | 0.579 | 0.046 | 0.310 | 0.041 | 0.362 | 0.027 | 0.545 | 0.054 | 0.251 |
| **Measuring time of thyroid hormone** |  |  |  |  |  |  |  |  |  |  |  |  |  |
| 1^st^ trimester | 1187 | 0.156 | <0.001 | 0.129 | <0.001 | 0.063 | 0.014 | 0.065 | 0.072 | 0.064 | 0.012 | 0.056 | 0.033 |
| 2^nd^ trimester | 1115 | 0.025 | 0.467 | 0.041 | 0.237 | 0.057 | 0.089 | 0.011 | 0.032 | 0.099 | 0.003 | 0.127 | <0.001 |
| **Neonatal gender** |  |  |  |  |  |  |  |  |  |  |  |  |  |
| Male | 1709 | 0.175 | <0.001 | 0.168 | <0.001 | 0.052 | 0.061 | 0.065 | 0.019 | 0.092 | 0.001 | 0.111 | <0.001 |
| Female | 1501 | 0.043 | 0.161 | 0.029 | 0.356 | 0.055 | 0.068 | 0.060 | 0.048 | 0.052 | 0.086 | 0.052 | 0.093 |
| **Delivery modes** |  |  |  |  |  |  |  |  |  |  |  |  |  |
| Vaginal | 2299 | 0.155 | <0.001 | 0.142 | <0.001 | 0.051 | 0.023 | 0.064 | 0.005 | 0.107 | <0.001 | 0.084 | <0.001 |
| Cesarean/ triceps | 912 | 0.113 | 0.002 | 0.102 | 0.005 | 0.064 | 0.073 | 0.073 | 0.042 | 0.062 | 0.083 | 0.100 | 0.006 |
| **Delivery weeks** |  |  |  |  |  |  |  |  |  |  |  |  |  |
| ＜37 | 129 | 0.000 | 0.998 | 0.052 | 0.610 | 0.091 | 0.357 | 0.106 | 0.284 | 0.135 | 0.181 | 0.207 | 0.042 |
| ≥37 | 3082 | 0.146 | <0.001 | 0.137 | <0.001 | 0.053 | 0.006 | 0.066 | 0.001 | 0.095 | <0.001 | 0.090 | <0.001 |

Analyses were conducted by multivariable linear regression models by enter methods with adjusted covariates including maternal age at pregnancy(y), parity, 1^st^ degree relatives of thyroid diseases(yes or no), education (less or equivalent to primary school, middle school, above high school), pre-pregnancy body mass index(kg/m^2^), body weight gain during pregnancy (kg), measuring time of thyroid hormones (gestational weeks); delivery mode(cesarean delivery/forceps, vaginal delivery), gestational weeks of delivery (gestational weeks), gender of infant (male or female), birth weight (kg). If the subgroup analysis was conducted for the stratified variable, this variable was excluded in the multivariable regression model. Abbreviations: BMI, body mass index; GDM, gestational diabetes mellitus; THs, thyroid hormones; FT3,free triiodothyronine; FT4,free thyroxine; TFQI, thyroid feedback quantile-based index; TT4RI, thyrotrophic T4 Resistance Index. TFQI=cdfFT4-(1-cdfTSH); TSH index=lnTSH (mIU/L) + 0.1345×FT4(pmol/L)；TT4RI=FT4 (pmol/L)×TSH(mIU/L).
